# The Progress of Therapeutic Vaccination with Regard to Tuberculosis

**DOI:** 10.3389/fmicb.2016.01536

**Published:** 2016-09-28

**Authors:** Pere-Joan Cardona

**Affiliations:** Unitat de Tuberculosi Experimental, Universitat Autònoma de Barcelona, CIBERES, Fundació Institut Germans Trias i PujolBadalona, Spain

**Keywords:** *Mycobacterium tuberculosis*, vaccines, *Mycobacterium w*, *Mycobacterium indicus pranii*, *Mycobacterium vaccae*, *Mycobacterium manresensis*, H56, neutrophils

## Abstract

A major problem with tuberculosis (TB) control is the long duration of drug therapy–both for latent and for active TB. Therapeutic vaccination has been postulated to improve this situation, and to this end there are several candidates already in clinical phases of development. These candidates follow two main designs, namely bacilli-directed therapy based on inactivated -whole or -fragmented bacillus (*Mycobacterium w* and RUTI) or fusion proteins that integrate non-replicating bacilli -related antigens (H56 vaccine), and host-directed therapy to reduce the tissue destruction. The administration of inactivated *Mycobacterium vaccae* prevents the “Koch phenomenon” response, and oral administration of heat-killed *Mycobacterium manresensis* prevents excessive neutrophilic infiltration of the lesions. This review also tries to explain the success of *Mycobacterium tuberculosis* by reviewing its evolution from infection to disease, and highlights the lack of a definitive understanding of the natural history of TB pathology and the need to improve our knowledge on TB immunology and pathogenesis.

## Introduction: understanding TB induction

Tuberculosis (TB) is still a major threat for humankind and is by far the most successful disease caused by an infectious agent ever. Indeed, it has been calculated that TB has already caused 1,000,000,000 deaths over the last 200 years alone (Paulson, 2013). Furthermore, TB is a condition highly affected by stigma. Shame and blame keep affected individual patients and society as a whole from putting TB high on the political and scientific agenda (Chang and Cataldo, 2014). TB still causes 1.5 million deaths and 10 million new cases per year (World Health Organization, 2015). In fact, it is calculated that a third of humankind already has a latent tuberculosis infection (LTBI; Dye et al., 1999; Dheda et al., 2015). After exposure to an active case of TB–especially, intensive and durable exposure to a highly infectious index case with cavitary TB and poor cough etiquette- LTBI may ensue; and of those individuals subsequently latently infected, around 10% will develop overt TB in subsequent years. Young individuals and those with reduced protective immunity stand the highest chance of developing TB following exposure; pregnant women, those with HIV coinfection, but to a leser degree also those with diabetes, etc. (Trauer et al., 2016). Furthermore, LTBI can last for a lengthy period, although the exact period remains a matter of controversy. Some authors, for example, have claimed that “once infected always infected” based on the “unitary concept” developed by Stead in 1967 (Stead, 1967; Cardona, 2015). Others, in contrast, reduce the magnitude of the infection to a limited, although relatively long, period of time, i.e., around 10 years (Cardona and Ruiz-Manzano, 2004).

Non-replicating (NR) bacilli are the responsible for this phenomenon as they arise due to the environmental stress caused by the immune response or chemotherapy itself (Wayne and Sohaskey, 2001). This stress causes a drastic change in the metabolism of the bacilli to become NR, a fact that makes them less vulnerable to chemotherapy or immune responses (Baer et al., 2015). As such, NR-*Mycobacterium tuberculosis* (NR-Mtb) are directly responsible for the extraordinary length of the chemotherapy against latent and active TB (Mitchison, 1965).

Therapeutic vaccines appear in this scenario where there is an urgent need for a new tool to shorten the chemotherapy treatment.

### The host scenario: alveoli are designed for gas exchange

It is not the aim of this article to extensively review the natural history of TB. However, it is very important to provide an overview of this disease in order to understand the battlefield on which infection takes place. In the case of *M. tuberculosis* (Mtb), this is especially important, as it targets one of the weakest links in the human immune system, namely the alveolar macrophage (AM).

**Figure 1:**
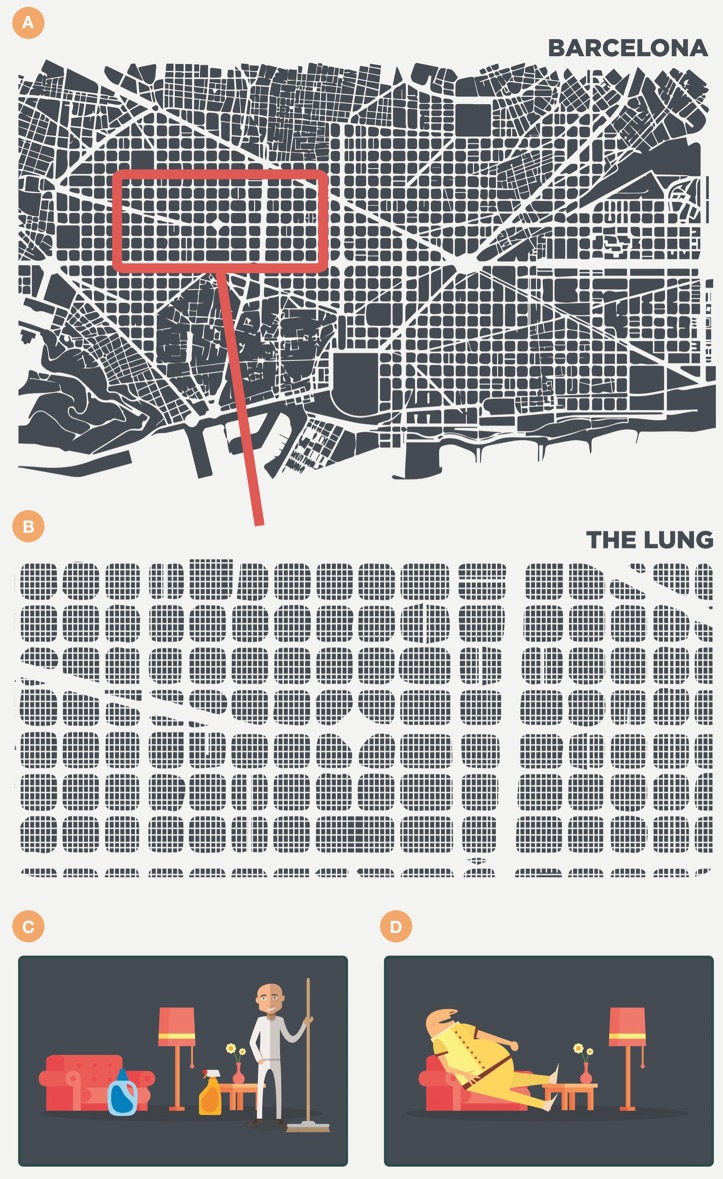
**Anthropomorphic view of the alveoli and the scenario in the lung**. The lung is like a big city (i.e., Barcelona) of around 625 km^2^, filled with 250 million of 25 m^2^ rooms **(A,B)**. Each room contains a cleaner—the alveolar macrophage **(C)**—that removes every particle that enters with the air every 6 s. Finally, however, this cleaner becomes filled with lipid inclusions, forms a foamy macrophage, becomes useless and is finally drained out of the room **(D)**.

Contrary to the normal, somewhat anthropocentric-type view, the AM is not some sort of police officer constantly on the look-out for pathogens and ready to deliver its antigens to trigger a fast and strong immune response. Alveoli are designed for gas exchange and have a very delicate structure, which is why we should perhaps consider the AM to be a highly specialized cleaner that must keep the space assigned to it clean (Figure 1). Following this anthropocentric approach, we can imagine this cleaner in a 25 m^2^ room, the door of which opens every 6 s to allow the entry of external air along with large quantities of dust and pathogens.

Figure 1 illustrates this by showing the lung as a large city, such as Barcelona (Catalonia), filled with 250 million 25 m^2^ rooms. This illustrates roughly the low range of the alveoli number in adults (Ochs et al., 2004) and, consequently, the same number of AMs, the most abundant antigen-presenting cell in the alveolar spaces (Guth et al., 2009), as it is logical to infer that each alveolus is occupied by a single AM (Suarez et al., 2012; Peake and Pinkerton, 2015). In this scenario, of healthy lung parenchyma, the presence, and role of dendritic cells (DCs) in the alveoli is very limited, if any, as they are present in very low numbers in the parenchyma as a whole (around 2%) and thus essentially absent in the healthy alveolar space. Moreover, even if present, AMs tend to suppress their activity (Landsman and Jung, 2007; Linton and Thoman, 2014). Consequently, there is a very particular scenario in which the conductor (the AM) is very reluctant to trigger any inflammatory response. This results in a two important delays in immunological surveillance, namely in communication level with the lymph node for the antigenic presentation; and in the attraction of specific lymphocytes that are able to activate the infected AMs.

**Figure 2:**
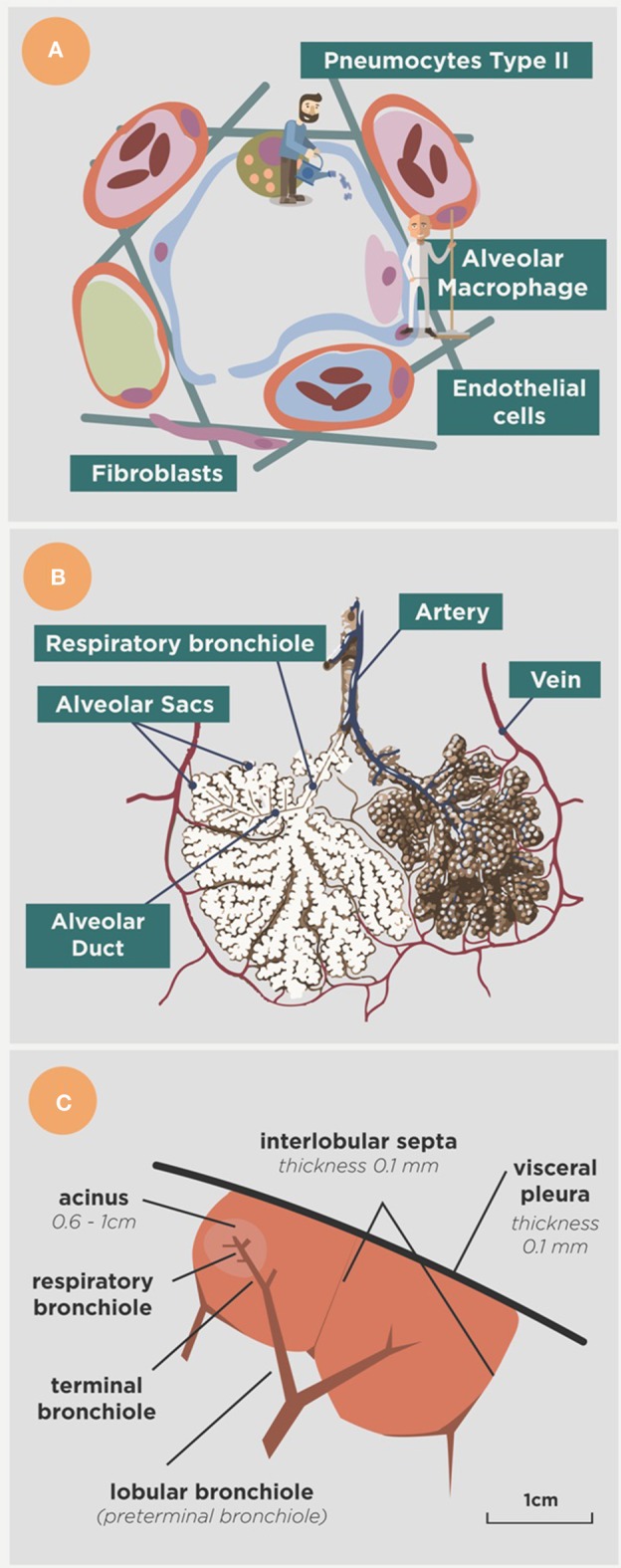
**Anatomical approach to the alveoli. (A)** Shows the main actors in the alveoli, namely the alveolar macrophage (the cleaner), the type II pneumocytes (the gardener) that form the surfactant, the epithelial cells that limit the space and the endothelial cells that limit the capillary net. Arterial vessels are shown in red, venous vessels in blue and lymphatic vessels in green. Fibroblasts produce the collagen fibers that structure the space. **(B)** Shows the organization of the alveoli in the alveolar sacs and acinus, and **(C)** provides a lower view showing the acinus in the context of the secondary lobe, surrounded by the interlobular septae net connected to the visceral pleura (modified from Webb, 2004).

The alveoli have another important characteristics, namely that they are mobile as they must be inflated and deflated in order to allow the entry of air (Macklem, 2005). This is possible because of the presence of a surfactant, produced by type II pneumocytes, which provides the surface tension required to avoid the collapse of the alveolus, which has a diameter of around 300 μm. At the same time alveoli must be sealed to ensure a lack of contact with the plasma transuded from the capillary network as this would constantly vary the surface tension (Suarez et al., 2012). This means that no antibodies can reach the alveoli, even when there is a minimal space between the capillary and the alveoli in order to allow gas transfer (Figure 2). Fortunately, the surfactant also has a micobactericidal activity (Arcos et al., 2011) and can contribute to the defense, along with some activity of AMs themselves, as a result of the phagocytosis of previously cleaned particles (that can also be detrimental) or a protective genetic background (Abel et al., 2014).

Overall, this means that in the context of a single AM infection, specific lymphocytes are not attracted, and that specific antibodies cannot reach the alveoli, unless a strong inflammatory response is triggered to disturb this equilibrium. This is the perfect scenario for Mtb infection and therefore makes the design of a prophylactic vaccine rather difficult, as has been noted recently (Lalvani et al., 2013; Kaufmann et al., 2015).

### Latent tuberculosis infection (LTBI): triggering the immune response

**Figure 3:**
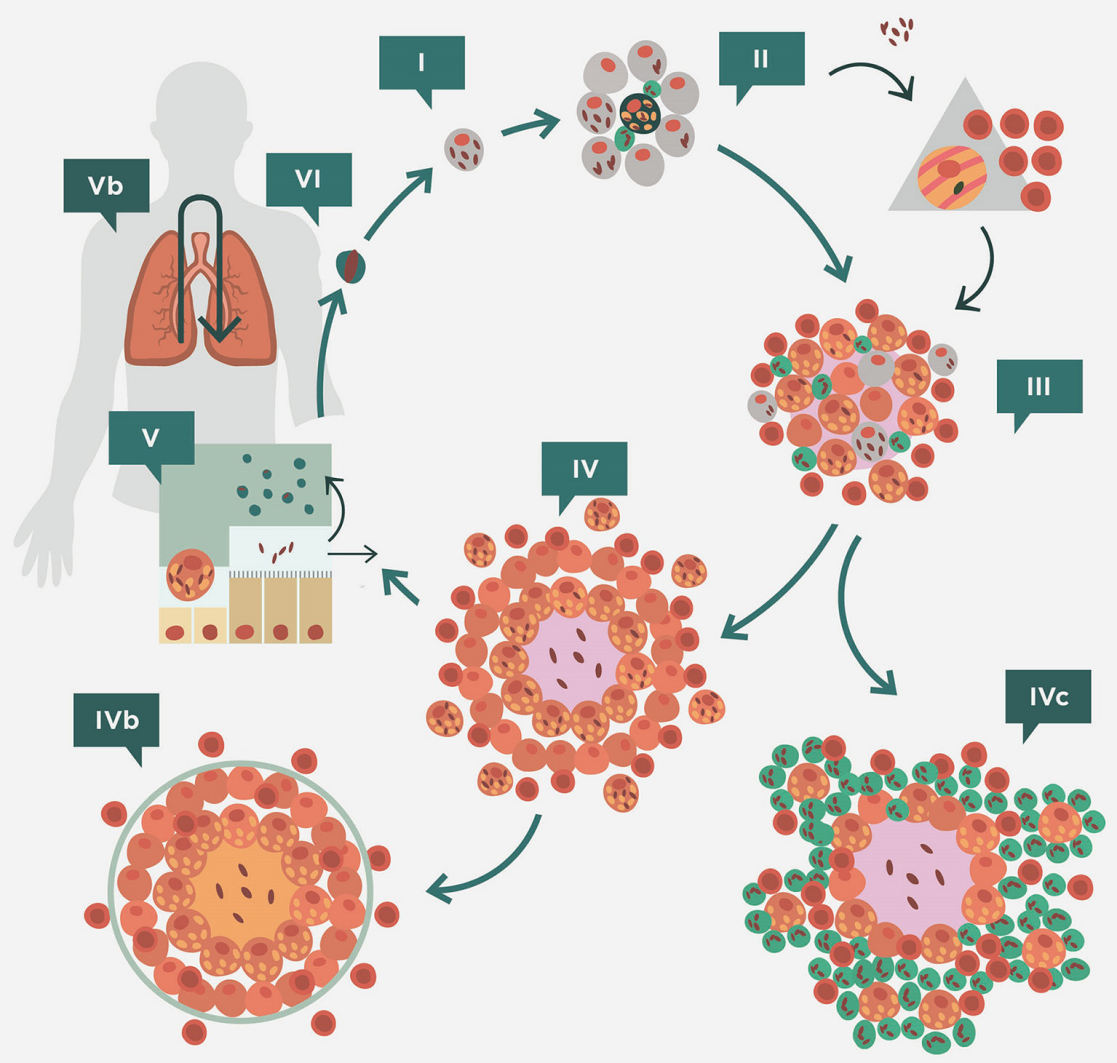
**Life cycle of ***Mycobacterium tuberculosis*** in the lungs according to the dynamic hypothesis**. After transmission via aerosol, *M. tuberculosis* settles in the alveoli. (II) *M. tuberculosis* grows inside alveolar macrophages (AM), causing their necrosis. These cells cause a weak, if any, inflammatory response and the bacilli released are simply phagocytosed by neighboring AMs, causing no lesion. This “unicellular” phase (I) allows the bacilli complete freedom to constantly generate new infectious foci, even in those hosts that have optimal cellular immune responses. This is because of the lack of antibodies in the alveoli to inactivate the bacilli and the fact that infected AM do not induce a sufficient inflammatory response to be detected by specific lymphocytes. Once the inflammatory response is sufficiently intense due to the large number of neighboring infected AMs, the alveoli break their sealed nature, thus allowing the entry of cells from the capillary net and the drainage of the bacilli toward the lymphatic vessels and regional lymph nodes, where antigenic presentation and lymphocytic proliferation take place (II). These lymphocytes are attracted to the inflammatory foci, where they activate the infected AM and destroy the bulk of the bacilli (around 90%); the survivors become non-replicating (NR-Mtb) and rest inside activated AMs or necrotic tissue (III). Once bacillary growth has been controlled, the “cleaning” phase starts. This phase is characterized by phagocytosis of the necrotic debris by the activated AMs, which retain even more NR-Mtb and become “foamy” by accumulating cellular debris (IV). These foamy macrophages are then progressively drained with the alveolar fluid toward the bronchi, where they are destroyed. The bacilli contained therein can pass into internal aerosols (V) and are able to reinfect tissue (VI), although they are mainly drained toward the intestinal tract (VI). This cycle can be interrupted by encapsulation, which isolates the granuloma. This process occurs as a result of the interlobular septae, which contain fibroblasts that are very sensitive to the mechanical changes caused by intraparenchymal lesions (IVb). Some hosts can develop an intense neutrophilic response, which is the origin of cavitated lesions (IVc). Version from Vilaplana and Cardona (2014).

Figure 3 shows the interaction between Mtb and the immune response in the scenario described above. This interaction defines a cycle in which a fine balance is established between the host and Mtb. The bacilli initially grow in the AM. This growth is very slow (every 24 h) and may therefore delay the induction of danger signals from the AM (Kimura et al., 2014). Depending on the strain, Mtb has a greater or lesser ability to avoid apoptosis of the infected AM, a process that could arrest the inflammatory response and limit bacillary growth (Lee et al., 2006). Indeed, a necrotic AM favors supplemental growth of the bacilli, thus increasing the bacillary load for the new incoming or for the neighboring AMs. An efficacious inflammatory response is triggered once sufficient AMs have been infected, thus allowing the entry of PMNs and monocytes from the capillary that start to build a granulomatous lesion. As a result of this bypass, the seal of the alveoli is broken and the bacilli can reach the interstitial space and be drained by the lymphatic vessels toward the lymph nodes (Chackerian et al., 2002). Once it reaches the lymph nodes, Mtb infects the local macrophages, thereby initiating lymph node infection; and the dendritic cells (DCs), which start the process of epitope presentation and specific Th1-lymphocyte proliferation (Vilaplana et al., 2014).

**Figure 4:**
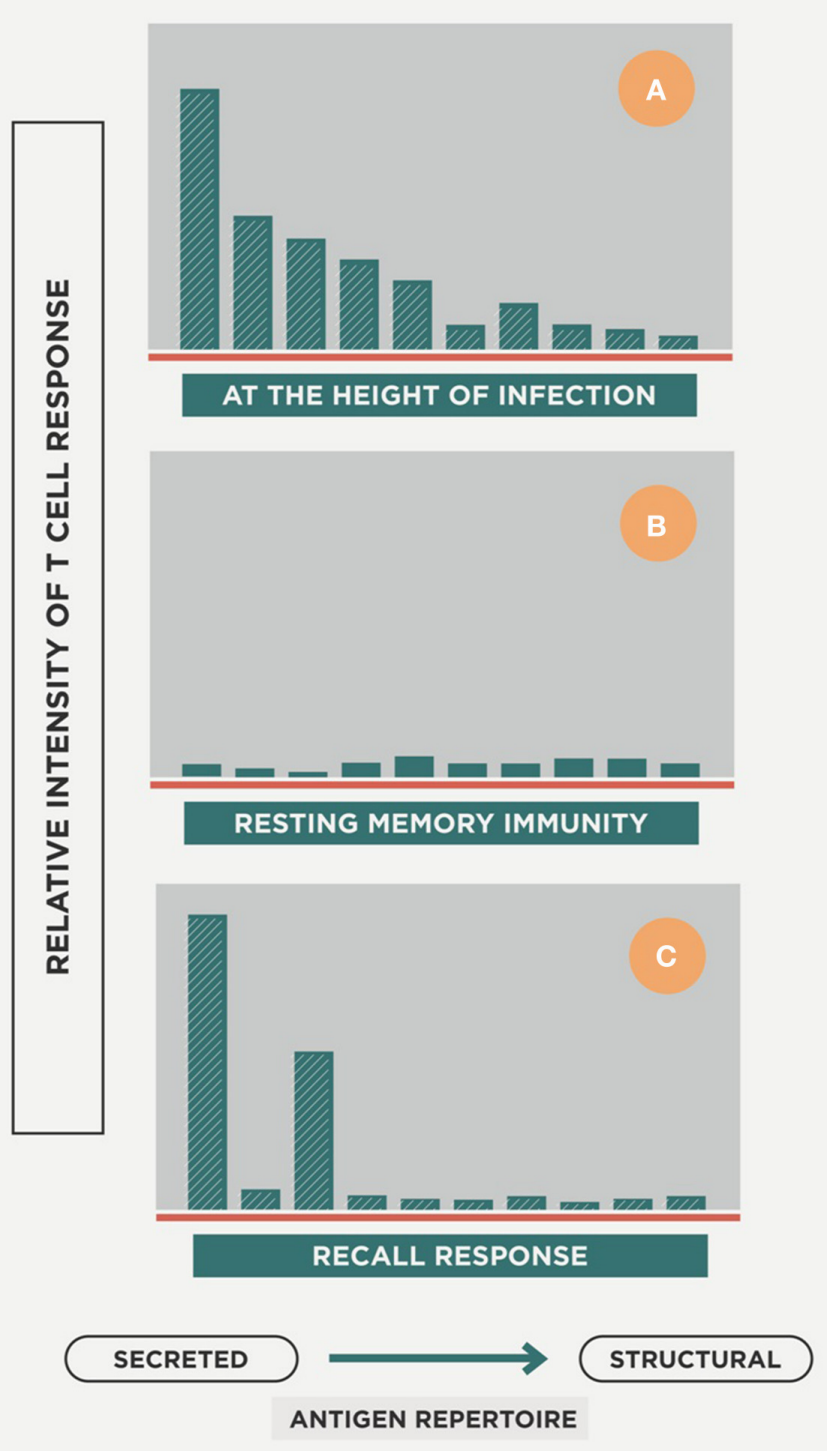
**Host response repertoire against Mtb infection**. The picture illustrates how, at the onset of infection, a wide-ranging cellular response is induced against a large epitope repertoire that mainly includes secreted antigens but also structural ones **(A)**. Once the acute phase is controlled and there is a majority of non-replicating Mtb (NR-Mtb; Muñoz-Elías et al., 2005), the immune response decreases while maintaining the memory status **(B)**. Once reinfection occurs, the immune response is triggered but essentially only against secreted antigens **(C)**. Modified from Andersen (1997).

Specific lymphocytes are triggered to a broad spectrum of epitopes, mainly those secreted by replicating Mtb (R-Mtb; Andersen, 1997; Figure 4), and are attracted to the granuloma to activate the infected AMs. Meanwhile, DCs are produced in the granuloma to provide feedback to the lymph node on the need for more lymphocytes at the infected site (Harding et al., 2015).

### LTBI: a constant reinfection process

The AM activation by Th1 lymphocytes causes Mtb destruction, but also generates NR-Mtb, which are mainly immersed in the intragranulomatous necrosis generated by the bacilli and the inflammatory response. These cellular debris together with the NR-Mtb are phagocytosed by old activated AMs that then become foamy macrophages (FMs; Cáceres et al., 2009; Russell et al., 2009), which are finally drained toward the bronchial escalator. FMs can be destroyed in this drainage, and the NR-Mtb have the chance to form part of the aerosols induced in the bronchioles, thereby again infecting the parenchyma. However, the majority are drained toward the upper portions of the tree, the pharynx, and are then swallowed into the gastrointestinal tract (Cardona, 2009). This is a very efficient way to control Mtb infection, with the immune response only being focused against R-Mtb; whereas NR-Mtb, which are more resistant to the stress (Dutta and Karakousis, 2014), are simply drained away. As the alveolar spaces are smaller in the murine lung, FM get trapped and are easy to see at the periphery of the granuloma (Cardona et al., 2003; Cáceres et al., 2009). This dynamic paints a scenario in which both R-Mtb and NR-Mtb can be found in the same lung, as described before (Muñoz-Elías et al., 2005). This is possible because of the presence of Mtb resuscitation-promoting factors that allow NR-Mtb to revert to R-Mtb (Mukamolova et al., 1998).

Coming back to Figure 4, upon reinfection the immune response is increasingly directed toward the antigens secreted by R-Mtb, a process that helps the survival of NR-Mtb, as they remain hidden from immune surveillance (Andersen, 1997).

Interestingly, a majority of Mtb strains are able to trigger a Th1 response and to provide a protective immune response, as good as that induced by BCG (Mollenkopf et al., 2004); although some exceptions have been reported (Manca et al., 2016). It is expected that the reinfection process is a common feature in high incidence countries. This means that there is a constant boost of the immune response against R-Mtb, which explains why the usefulness of prophylactic vaccines, such as the Bacillus Calmette Guerin–BCG-, in these countries is mainly reduced to providing an advantage to the host when faced with its very first Mtb challenge, i.e., in neonates. Specifically, it reduces the onset of the immune response by about 5 days (Jung et al., 2005; Cardona and Vilaplana, 2014), but does not prevent the infection itself.

### LTBI: the role of the local milieu

It is interesting to note that the lesion caused is usually based on the presence of AMs, some rafts of PMNs, some intragranulomatous necrosis, and is surrounded by lymphocytes (the “doughnut” appearance). This is because granulomas, where the majority of AMs are activated, tend to inactivate the entry of new lymphocytes. This phenomenon has been well-described in mice and guinea pigs and appears to arise as a consequence of different mechanisms, including the competition to obtain arginine, direct suppression, or apoptosis, amongst others (Zhang and McMurray, 1998; Ríos-Barrera et al., 2006; Jeevan et al., 2007).

The lung parenchyma in large mammals is structured into 2i lobes that build a network of interlobular septae which connects the whole parenchyma with the visceral pleura and transmits the mechanical force induced by the diaphragm to expand it (Webb, 2004; Figure 2). This network allows the alveolar walls to be as thin as possible to facilitate gas exchange (Peake and Pinkerton, 2015), as they do not have to transmit the strength of the whole lung expansion. This fact is very relevant in big mammals as they have a larger lung parenchyma to expand (Peake and Pinkerton, 2015). These interlobular septae are very sensitive to any minimal mechanical change, such as the minimal lesions induced in LTBI (with a diameter of around 0.5 mm), and are able to encapsulate and isolate them from the healthy parenchyma within about 10 days (Gil et al., 2010). This mechanism is very important as it helps to stop the drainage of NR-Mtb and finally kills them as a result of a poly-stress mechanism based on hypoxia, high pH, starvation, and finally by the hyperosmosis caused by the accumulation of calcium (Gil et al., 2010).

### Why does active TB occur? anatomy of the lung and quality of the lesions for a perfect storm

Looking at the consequences of LTBI, it is clear that it causes a negligible health impact. Problems arise, however, when the lesions are not well-controlled and become bigger, thereby progressively hampering patient health as a result of destruction of the parenchyma. The main challenge is to understand the mechanism that allows a tiny lesion of less than 1 mm, in the case of LTBI, to become a lesion with a diameter of around 10 mm (which is the minimum required for detection in a chest X-ray; Andreu et al., 2004).

Considering the dynamics of Mtb, it is hard to believe in the concept that a bacillus from an old lesion resuscitates and is able to trigger a massive infiltration that leads to liquefaction of the tissue and extracellular growth of the bacilli, which is the current dogma (Grosset, 2003). In fact, there is considerable experimental evidence that R-Mtb must constantly attempt to resettle in the parenchyma to induce new lesions otherwise its fate is to be drained as illustrated in Figure 3 (Cardona and Ivanyi, 2011).

**Figure 5:**
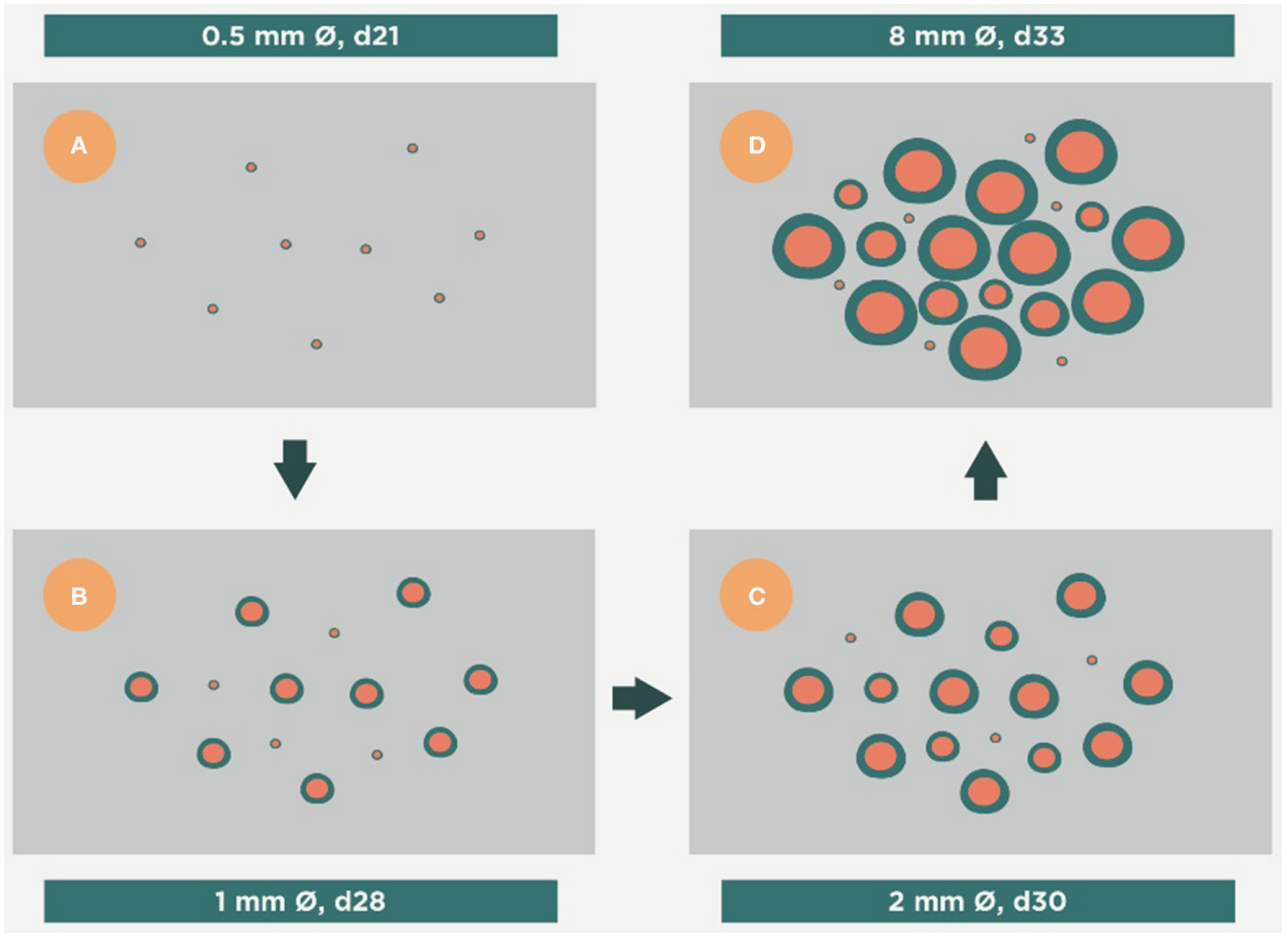
**Evolution of the infection toward active disease**. An excess of Th17 allows massive infiltration of the lesions by PMNs. This causes a rapid growth in their size and the induction of secondary lesions (daughter lesions). Finally, these lesions coalesce, which results in the evolution of small granulomas (<1 mm) to large infiltrations that can liquefact and cavitate, thus exceeding the ability of the interlobular septae to encapsulate them. Time period from **(A–D)**, days 21, 28, 30 and 33 respectively.

Recently, an explanation has been provided by the study of a murine mode of TB in the mouse strain C3HeB/FeJ, which develops human-like lesions (Marzo et al., 2014). This model supports the idea that the tiny lesions of LTBI have only a narrow window of opportunity to generate large lesions, otherwise they are quickly encapsulated and become unable to do so. Essentially, this opportunity comes as a result of two factors (Figure 5). The first of these is infiltration by PMNs, which generate Neutrophilic Extracellular Traps (NETs) to stop Mtb replication (Ramos-Kichik et al., 2009). However, this induces the opposite effect as NETs becomes a platform that allows extracellular growth of Mtb. This is very interesting because extracellular growth was usually linked to the presence of liquefaction (Grosset, 2003) and was not thought to occur before. As such, the presence of NETs helps rapid enlargement of the lesion. In fact, the relation between granulocyte accumulation and TB progression was described before in TB-highly-susceptible (Lyadova et al., 2010) and in several KO immunosuppressed mice (Gil et al., 2006). The crucial role of this extracellular bacillary population at the beginning of the transition from infection to active TB is that opens up the possibility for an antibody-based therapeutic vaccine that would help the clearance of this population and stop progression toward active TB. Indeed, passive antibody therapy has provided excellent results as regards controlling the evolution of lesions in an experimental model with SCID mice, which have an impaired B- and T-cell response and build the response against Mtb with NK cells and PMNs (Guirado et al., 2006).

The second factor is the coalescence of different yet closely related lesions, including new ones, as a consequence of local drainage (Prats et al., 2016). Both these factors mean that 10 mm lesions can be induced in about 10 days (Marzo et al., 2014; Vilaplana and Cardona, 2014).

Curiously, this phenomenon follows the same physical processes as soap bubble formation, thus allowing an “*in silico*” model, known as the “bubble model” (Prats et al., 2016), to be developed to help to understand the induction of active TB.

In humans, TB lesions tend to accumulate in the upper lobe (Dock, 1954). The most favored hypothesis relates this tropism with the higher oxygen pressure in this region that might favor Mtb growth. This is related to the gravitational force that causes the lung to hang from the upper side of the costal thorax, thereby favoring a higher intraalveolar pressure that reduces capillary circulation and gas exchange and resulting in a higher partial higher oxygen pressure.

**Figure 6:**
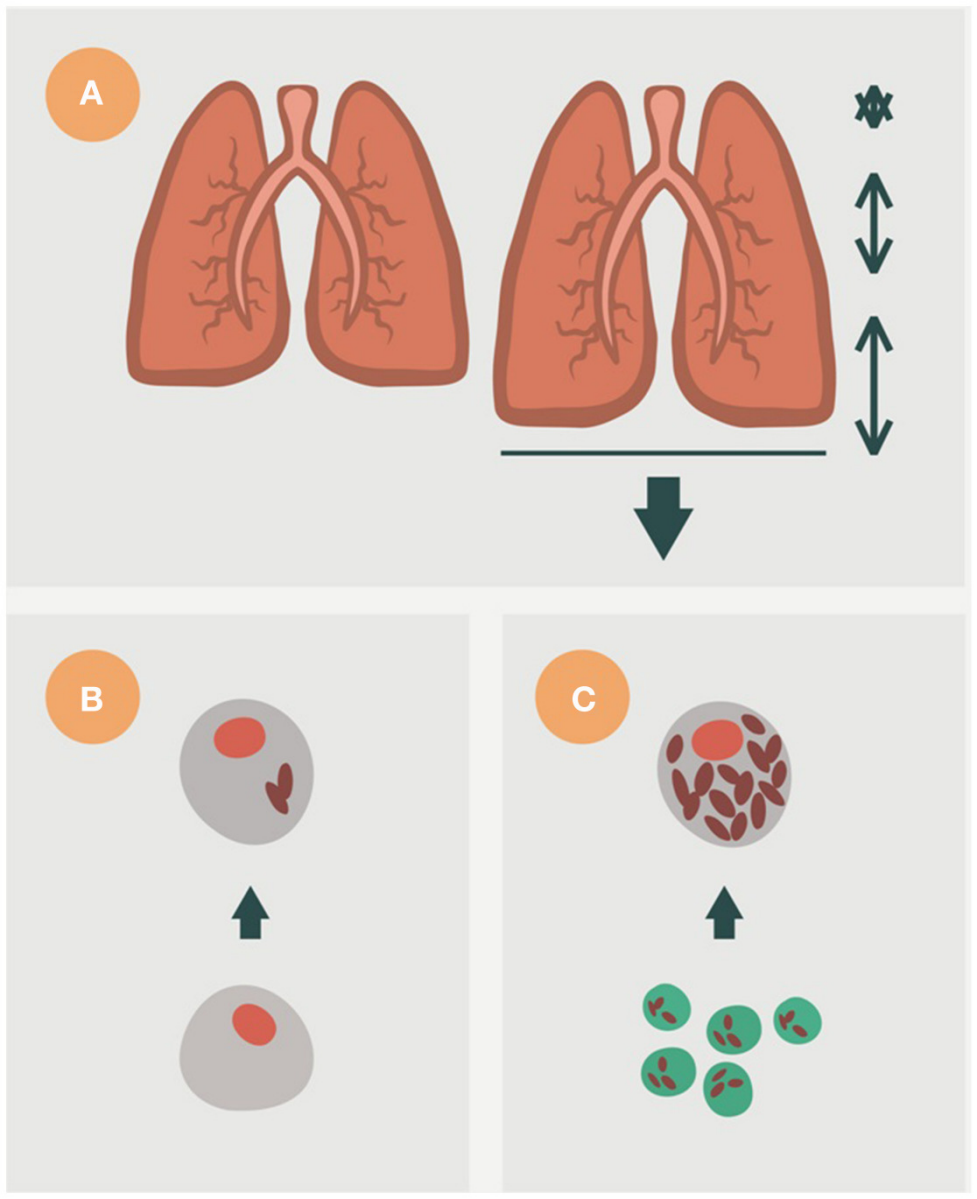
**Importance of breathing amplitude in the lung, for the induction of active TB**. In mammals, breathing in, or inhaling, occurs due to the contraction and flattening of the diaphragm, a domed muscle that separates the thorax and abdomen **(A)**. This action increases the space of the alveoli, although not homogeneously. The lobes of the base experience the largest amplitude, whereas the upper lobes experience almost no movement. This fact has a major consequence, namely a very important drainage of bacilli after destruction of the alveolar macrophage (AM) toward neighboring alveoli in the base, with almost none at the upper lobes. This means that new AMs face a lower bacillary load in the base than in the upper lobes **(B)**. The increase of the bacillary load stimulates necrosis and neutrophilic infiltration **(C)**. This hypothesis can explain why the evolution toward active TB mainly occurs in the upper lobes.

The mechanical force required to inflate the lungs is generated by the diaphragm at the base of the lung, which moves the parenchyma toward the abdominal cavity (Figure 6), thus resulting in a low breathing amplitude in the upper lobes that also favors hyperoxia (Gurney, 1991; Guo et al., 2011). Furthermore, it also causes a lack of local particle drainage in the upper lobes and, therefore, an increase in the bacillary load faced by the AM (Figure 6). This favors a response similar to that triggered against extracellular bacilli, firstly stimulating PMN infiltration (Gil et al., 2006; Gan et al., 2008) and then an immune response dominated by the Th17 response, which also favors infiltration with PMNs (Korn et al., 2009; Torrado and Cooper, 2010). An “*in silico*” model has recently been built to illustrate this process (Cardona and Prats, 2016).

**Figure 7:**
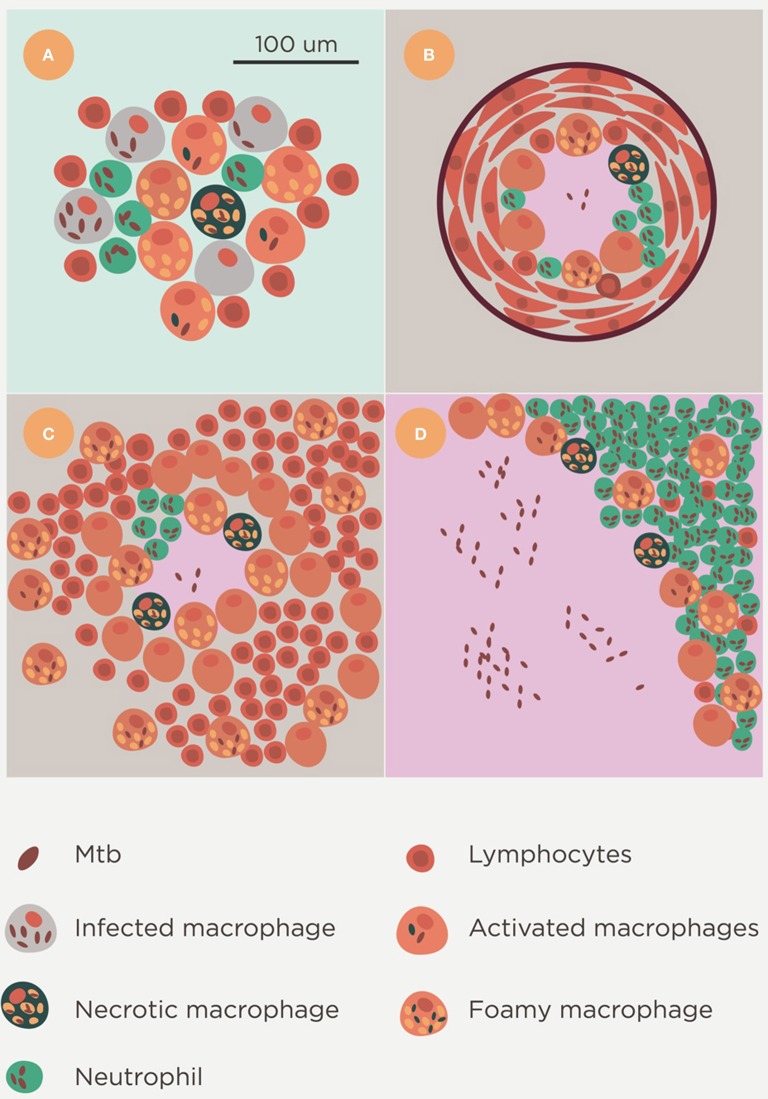
**Lesion spectrum of Mtb infection**. Histological evolution after Mtb infection. Initial foci containing a mixture of neutrophils, macrophages and lymphocytes **(A)** are soon controlled by the encapsulation process and either calcify **(B)** or progress with constant reactivation of the bacillary load in foamy macrophages of the neighborhood. This is typical in the murine infection **(C)**. These lesions could fall into the category of proliferative lesions. Exudative lesions are characterized by the large-scale infiltration of neutrophils, a high bacillary burden, and the presence of massive necrosis **(D)**.

Two main lesions can be detected in pulmonary TB in humans: small, proliferative lesions that harbor a low bacillary load and mainly comprise epithelioid cells and are well-structured and fibrous, and exudative lesions, in which PMN infiltration is the major component and pus (liquefaction) may also be present (Figure 7; Prats et al., 2016). The evolution of TB results in a kind of patchwork scenario, comprising proliferative and exudative lesions, that is dependent on local endobronchial dissemination (Medlar, 1955). Other factors, such as a certain genetic tendency of AMs to trigger a stronger inflammatory response against Mtb or to induce more or less apoptosis, may also play a role, although which particular factor might have an influence has not been elucidated to date (Abel et al., 2014).

There is also extrapulmonary TB, which can be linked to immunosuppression in the case of meningeal or miliary forms, even though Mtb is able to infect any tissue. Extrapulmonary TB requires the entry of Mtb in the bloodstream (Krishnan et al., 2010). Although, some authors have claimed this to be due to the ability of Mtb to enter into epithelial cells and blood vessels, classically this has been linked to local disruption of the aberrant capillary net built around Mtb granulomas (Hunder, 1996); with larger lesions leading to a higher risk of dissemination (Medlar, 1955).

In summary, infection with Mtb clearly follows the “damage theory” paradigm (Casadevall and Pirofski, 1999; Cardona, 2010) in which both pathogen type and type of host' response triggered against the pathogen are key to induction of the disease.

## Therapeutic vaccines

**Figure 8:**
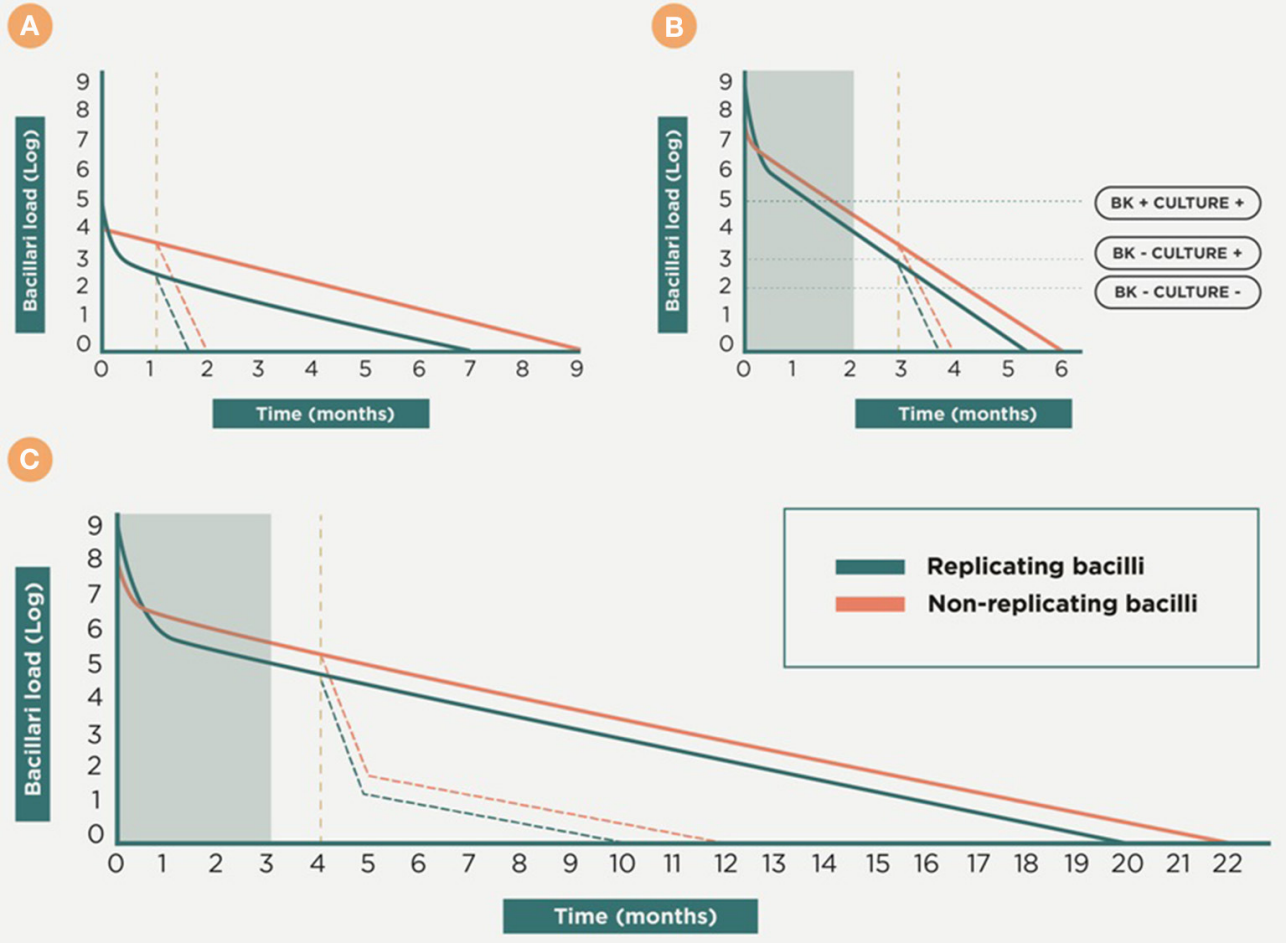
**Therapeutic vaccination as coadjuvant for chemotherapy**. This is a theoretical approach to the dynamics of the bacillary concentration during chemotherapy and the influence of vaccination as a coadjuvant. Initially, the early bactericidal activity induces a massive reduction in the bacillary load by killing replicating bacilli (R-Mtb). Subsequently, non-replicating bacilli (NR-Mtb) dominate and are the responsible for the reinfection process and the appearance of R-Mtb during chemotherapy (Muñoz-Elías et al., 2005; Cardona, 2006b). The purple dotted line shows a proposal of the time-point to introduce the therapeutic vaccination, once a drastic reduction of the bacillary load is obtained and the NR-Mtb becomes the majority population. From there, the evolution of R-Mtb (green) and NR-Mtb (red) is also drawn in dotted lines to show the added bactericidal effect. Panel **(A)** shows the treatment of LTBI with isoniazid. Panels **(B,C)** shows the treatment of active TB, considering one of the worse cases (i.e., with a bacillary load of 9 logs), in sensitive and multidrug resistant (MDR) TB, respectively. In both cases the gray box shows the initial intensive phase period of chemotherapy.

The use of “therapeutic vaccines” is an old strategy designed by Robert Koch himself (Cardona, 2006a) and received significant attention until the onset of chemotherapy. The fascinating story of the large-scale therapeutic used of the so called “tuberculins” has recently been reviewed by Vilaplana and Cardona (2010). Figure 8 shows a scenario in which a therapeutic vaccine can work together with chemotherapy. In essence, we expect the same phenomenon against latent and in active TB, with the therapeutic vaccine being used once there is a predominance of NR-Mtb and chemotherapy only being given to maintain sufficient tissue concentrations to stop regrowth of the bacilli. This is the moment to use a therapeutic vaccine, in order to achieve an extra bactericidal activity.

Two mechanisms of action can be defined. The first of these, which we can refer to as “bacilli-directed therapy,” increases the surveillance capacity against NR-Mtb by enhancing the cellular immunity to find those antigens linked to NR-Mtb that are hidden to the immune surveillance (Figure 4). In the case of the second, which is known as “host-directed therapy,” the objective is to modulate the inflammatory response in order to avoid extracellular growth of the bacilli fuelled by the accumulation of neutrophiles, or the Th2 response (or “Koch phenomenon”), that induce intragranulomatous necrosis.

### Bacilli-directed therapy: focusing on killing the bacilli

#### *Mycobacterium w (Mw)*, also known as *Mycobacterium indicus pranii* (MIP)

This strain is in the *Mycobacterium avium* complex (Alexander and Turenne, 2015) and was used heat-killed in initial attempts to develop a therapeutic indication in patients with borderline lepromatous or lepromatous leprosy to invigorate cell-mediated immunity. Patients receiving it exhibited rapid clinical improvement and improved histopathology of skin lesions, and it was found to significantly reduce the bacillary load (Talwar et al., 1990; Zaheer et al., 1993; Narang et al., 2005). Large quantities of data were obtained during clinical trials in India involving 80,000 patients receiving a treatment that combined chemotherapy with the administration of heat-killed *Mw* every 3 months.

In Mtb infection, the mechanism of action is based on the induction of a Th1 response and a reduction of Tregs (Das et al., 2015). This has been demonstrated in an experimental model in C57Bl/6 mice, with a significant decrease in bacillary load being achieved upon combining chemotherapy for 60 days with *Mw* vaccination every 2 weeks, with a total of five inoculations (Rawat et al., 2013). In addition, a combination of chemotherapy plus *Mw* also induced a reduction in bacillary load in an experimental guinea pig TB model (Rawat et al., 2013). In this case, the protective effect was related to an increase in CXCL10 and CXCL11 chemokines. Adjunctive therapy with Mw has been shown to be effective against multidrug resistant strains and has demonstrated its ability to stop extrapulmonar dissemination (Faujdar et al., 2011). More recently, in a trial involving 1400 patients with TB pericarditis (Mayosi et al., 2014) the administration of five injections of *Mw* over 3 months as an adjuvant to chemotherapy had no significant effect on the incidence of death, cardiac tamponade requiring pericardiocentesis, or constrictive pericarditis.

#### RUTI

**Figure 9:**
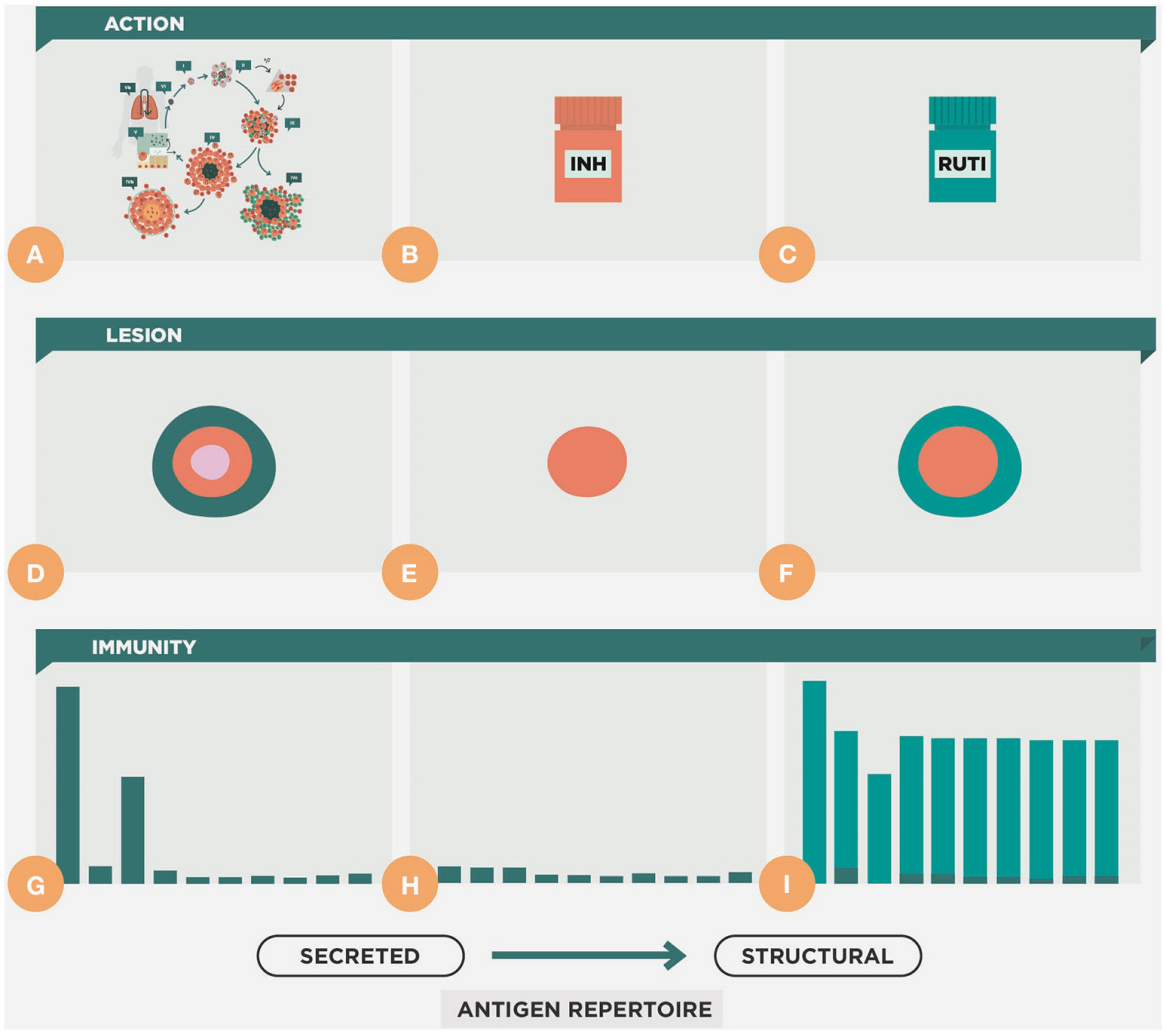
**Therapeutic approach of the RUTI vaccine**. This figure shows the natural evolution of the infection, with constant cycles of reinfection **(A,D,G)**. During chemotherapy there is a reduction of the lesion and the cellular immune response **(B,E,H)**. Upon vaccination with RUTI **(C,F,I)** there is an increase in the immune response that includes a wider repertoire, compared to that obtained with the usual reinfection process, which is more focused on the secreted antigens, as shown in Figure 4.

RUTI was the first therapeutic vaccine designed with the aim of killing NR-Mtb. This vaccine comprised fragments of Mtb cultured under stress conditions and the idea arose upon visualizing the drainage of NR-Mtb from the granulomas inside FMs (Cardona, 2006b). The aim was to induce an immune response that would be able to survey NR-Mtb. As we have noted previously, the immune response is mainly focused against R-Mtb antigens, therefore the question was how to change this. Firstly, this would require a short antibiotic treatment to kill the R-Mtb (Figure 9). This chemotherapy treatment would also be responsible for stopping lymphocytic proliferation and would start to disintegrate the granuloma and allow the entry of new, inactivated AMs or monocytes, thus making the granuloma a suitable site for further entry of a new repertoire of lymphocytes without the risk of being suppressed. It would also reduce the bacillary load to a minimum and would reduce the risk of the toxic “Koch phenomenon,” which was known to be the origin of the intragranulomatous necrosis (Gil et al., 2006). This risk can also be minimized by removing the majority of lipoarabinomannan (LAM) from the Mtb fragments. Due to its endotoxin-like nature, LAM was shown to be responsible for promoting intragranulomatous necrosis as a result of a “Schwartzman reaction,” which was linked to the origin of the “Koch phenomenon” (Cardona et al., 2001).

The criterion for the protective mechanism expected from RUTI (the GO/No-GO criteria) was to demonstrate the triggering of a broad response against R-Mtb and NR-Mtb antigens, and this was found to be the case. Once its bactericidal activity when administered after chemotherapy in murine and guinea pig models had been demonstrated (Cardona, 2006b), RUTI showed an ability to generate this polyantigenic response in 16 healthy volunteers (Vilaplana et al., 2010), and in HIV-positive and -negative patients with LTBI (Nell et al., 2014) after INH treatment for 1 month, in a phase II clinical trial (with 98 patients). In this case, a definitive dose was chosen to be more effective (25 μg) and only one shot was required to induce the desired response. Only local adverse events were detected. These were mainly non-severe, with a delayed presence of local self-resolved painless nodulation at 3 weeks post-vaccination on average. Abscessification was only detected in four cases. The frequency and severity of these adverse events were correlated with HIV-positive subjects, high vaccine dose and were more frequent after the second administration of the vaccine. After this trial, the RUTI production process changed to include a filtration step in an effort to reduce the nodulation at the inoculation site (Kaufmann et al., 2014).

#### H56

This is the proposal from the Statens Serum Institute and uses a fusion protein containing epitopes of ESAT-6 and Ag85B antigens, which are immunodominant in the secreted proteins of R-Mtb, plus the antigen Rv2660c, which is related to the NR-Mtb.

This vaccine demonstrated a consistent bactericidal effect in the Cornell-like model in mice, which combines chemotherapy once the chronic phase of the infection has been established in the C57BL/6 strain, with two or three inoculations of this vaccine (Aagaard et al., 2011). It shows a similar protection as the RUTI vaccine (Cardona et al., 2005) and also demonstrated its ability to protect against the progression from LTBI to active TB induced by the administration of anti-TNF antibodies, in an experimental model in cynomolgus macaques (Lin et al., 2012). Recently published data from the phase I clinical trial published recently show a strong Th1 response with a strong central memory phenotype in the 25 subjects enrolled. No serious adverse events were reported, although nine subjects (36%) presented transient cardiovascular adverse events (Luabeya et al., 2015).

### Host-directed-therapy: modulating the host reaction

#### Heat-killed *M. vaccae/M. obuense*

The idea of using heat-killed *Mycobacterium vaccae* came from the observation that some people from villages in Uganda where this species was abundant in the dust had some degree of natural protection against TB. The other pointer was the interference of environmental mycobacteria (EM) with the protection elicited by BCG. This issue was first addressed experimentally in mice by Brown et al. (1985), who added *M. vaccae* to drinking water for different periods of time before administering BCG. Upon testing the splenocytes for their proliferation capacity when stimulated with killed mycobacteria, oral administration was found to either enhance, mask or interfere with the protective effect of BCG. Meanwhile, two types of cellular responses against infections caused by intracellular pathogens were identified. The “Listeria-like” one, which is able to induce a cellular response without causing intragranulomatous necrosis, and the “TB-like” or “Koch phenomenon,” which is responsible for inducing this necrosis (Rook et al., 1981; Stanford, 1991). This response was linked to a predominance of Th1 or Th2 responses, respectively. In light of these findings, efforts were focused on the more controlled administration using parental immunization of heat-killed *M. vaccae* to convert the existing necrotizing response into a protective one (i.e., from Th2 to Th1; Stanford, 1991). This approach was first used to treat leprosy patients, and shortly thereafter was used in TB patients in several trials that have recently been summarized in the literature, showing no definitive results (Gröschel et al., 2014). Along similar lines, von Reyn's group had a positive experience in the DARDAR clinical trial in Tanzania, where the combination of several inoculations with heat-killed *M. vaccae* reduced TB definite cases by 39% in HIV-positive patients (von Reyn et al., 2010). Further genomic characterization has shown that the *M. vaccae* strain used by this group was actually *M. obuense* rather than *M. vaccae* (Lalvani et al., 2013). In 2011, a high yield and scalable GMP broth manufacturing process was developed in collaboration with Aeras. The vaccine (now DAR-901) is in Phase I since 2014, as is intended to be a booster vaccine for the prevention of TB in both HIV-infected and HIV-uninfected adolescents and adults primed by childhood BCG immunization (Kaufmann et al., 2014).

#### Heat-killed *M. manresensis*

This approach was developed after the observation of the key role of the Th17 response in the induction of active TB (Marzo et al., 2014). As Th17 lymphocytes and Tregs are in constant balance, this led to the idea of increasing Tregs in order to reduce the presence of Th17 lymphocytes. This could be done by inducing tolerance using the oral administration of low dose heat-killed mycobacteria. The role of regulatory T cells (Tregs) in TB has been controversial as they were initially thought to fuel progression toward active TB (Semple et al., 2013). However, recent reports seem not to support this idea (Green et al., 2010; Leepiyasakulchai et al., 2012), thus leading to a more neutral role in their interference against the Th1 response, and showing its protection against the development of active TB lesions.

Oral administration of heat-killed Mtb and BCG every day or 2 days was shown to delay the induction of active TB in a murine model in C3HeB/FeJ mice, in which “human-like” lesions can be reproduced (Cardona, 2015). This led to the search for an environmental mycobacterium in order to produce an affordable industrial product. As a result, the *fortuitum* complex was selected. As this bacillus is commonly found in drinking water, it can be classified as food supplement in regulatory terms. The strain used was obtained from a natural source (the river Cardener, which flows through the city of Manresa, Catalonia). Once the genome had been sequenced to satisfy regulatory requirements, it was realized that the strain was quite close to *Mycobacterium setense* but exhibited sufficient taxonomical differences to be considered a new species. As such, it was named *Mycobacterium manresensis* after the city of Manresa (Rech et al., 2015).

**Figure 10:**
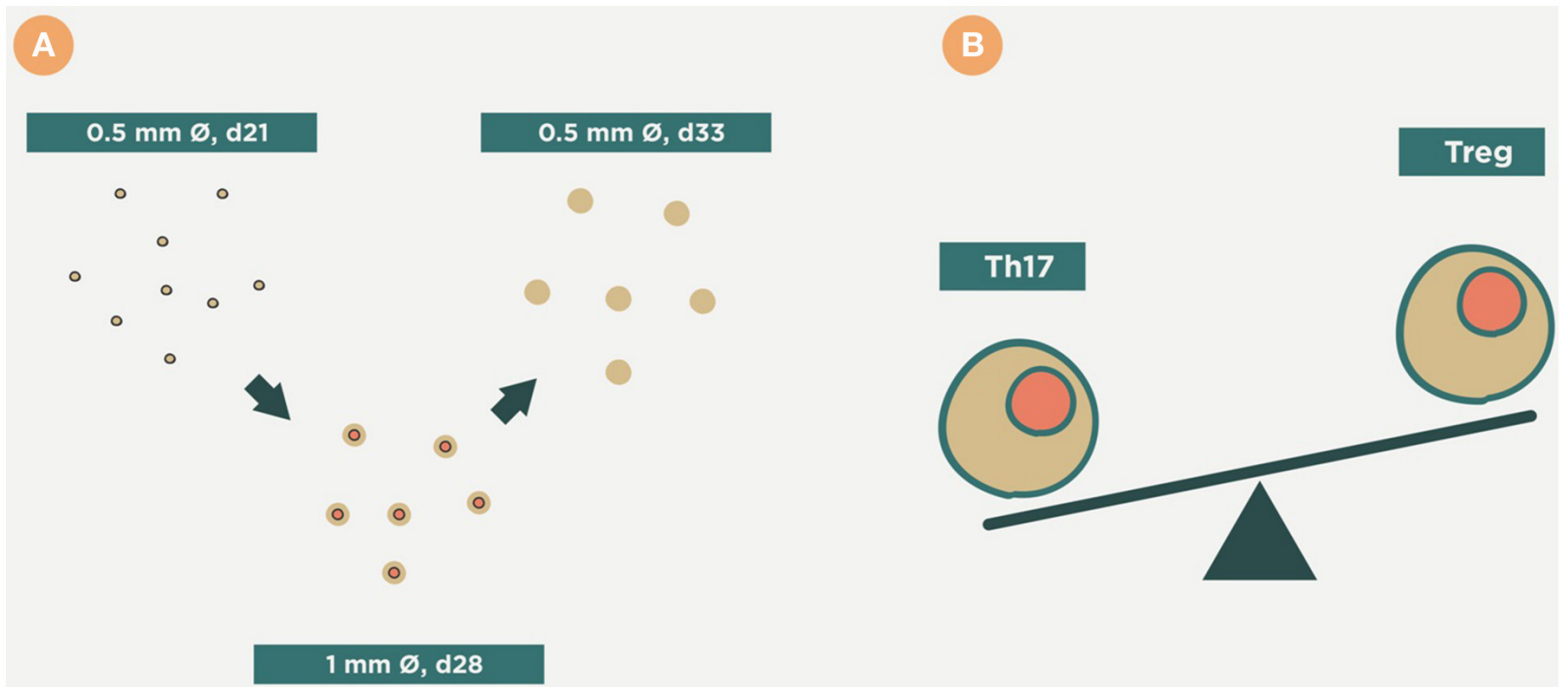
**Therapeutic approach of ***Mycobacterium manresensis*****. This picture illustrates how administration of *M. manresensis* increases the Treg response and results in a decrease in the Th17 response **(B)**. This stops the neutrophilic infiltration of the lesions, and stops their growth, and it has been speculated that this might provide sufficient time for them to become encapsulated **(A)**. This might avoid the progression toward coalescence, liquefaction, and cavitation.

Experimental data show that the oral administration of heat-killed *M. manresensis* is able to induce memory-specific Tregs and to delay the exaggerated inflammatory response that leads to the induction of active TB (Cardona et al., 2016). It has been speculated that this delay might provide sufficient time for the fibroblasts in the interlobular septae present in human lungs to encapsulate the lesions (Figure 10). The first clinical trial has demonstrated that daily administration of 10^5^ heat-killed *M. manresensis* (Nyaditum resae®, Manremyc sl, Manresa, Catalonia) for 14 days induces memory PPD specific Tregs, and has an excellent tolerability (Montane et al., 2014).

### Other therapeutic vaccines

Other promising candidates are still in the preclinical phase. Several DNA vaccines codifying mainly for the hsp65, Ag85A, and B antigens have been tested. Some of these have demonstrated their value in murine models, with or without chemotherapy and with a wide range of protection, as reviewed by Lowrie (2006). Recently, a group of therapeutic vaccines comprising different combinations of fusion proteins has provided very promising results. Thus, the “3-iron”vaccine candidate, which includes several iron-acquisition proteins (Shanley et al., 2014), has demonstrated a reduction in pathology and increased survival in the guinea pig model without the help of chemotherapy, although it was unable to reduce the bacillary load in the lung. Interestingly, this candidate showed this protection against highly virulent MDR strains. Similarly, ID93/GLA-SE, a candidate designed by the Reed group (Coler et al., 2013), is a combination of *M. tuberculosis* proteins associated with virulence (Rv2608, Rv3619, and Rv3620) or latency (Rv1813). ID93/GLA-SE has demonstrated its therapeutic value in combination with rifampicin and isoniazid in SWR/J mice and cynomolgus monkeys. This candidate elicits a robust Th1 response and is able to reduce the pulmonary bacillary load and increase the survival of the animals.

## Conclusion

Infection with Mtb clearly follows the “damage theory” paradigm in which interaction with the host is paramount for the induction of active disease. The fact that Mtb is able to grow inside the AM is clearly one advantage, but not the only one, as other pathogens are also able to do this. However, it is also able to induce necrosis inside these lesions. This favors additional growth of the bacilli and helps the progressive destruction of the parenchyma that leads to the induction of active TB.

Prevention of the infection itself seems to be controlled by some intrinsic mycobactericidal properties of the AM and, possibly, the properties of the surfactant. The immune response is not able to avoid reinfection because of the difficulty of attracting specific lymphocytes to isolated infected AMs and the lack of antibodies in the alveoli. Similarly, the induction of NR-Mtb once the immune response has been triggered makes very difficult to develop a chemotherapy that is able to eradicate them from the tissues in a short-period of time.

As such, there is an urgent need for therapeutic vaccines in the fight against TB, especially for those persons with LTBI. The current approach (6–9 months INH treatment) is very difficult to follow up for two reasons. Firstly, because of problems with compliance and toxicity, and secondly because reinfection is highly probable in high incidence countries once the treatment has finished, thus making this effort worthless. Equally, the presence of INH-resistant strains could potentially make this treatment highly ineffective. Although other shorter treatments are currently being proposed, they can still not be compared with an immunological intervention in terms of compliance.

Therapy for the disease itself is also of great importance, and in this regard, it would be highly significant to find an immunological approach to shorten treatment itself and to avoid relapse of the disease.

## Key points

1. The anti-infection capacity of the alveoli is very much conditioned by their main function: the gas exchange.
2. Mtb infects alveolar macrophages, cells with a dedicated cleaning task that do not elicit an inflammatory response which could alter their minimal architecture. The price of this, however, is to hamper antigen presentation and attraction of the cellular immune response.
3. Alveoli maintain a strong air-tightness to keep the appropriate surface tension, thus avoiding their collapse and allowing breathing mechanics. However, this prevents the entry of plasma and means no antibodies are present.
4. Mtb reinfection is always possible. Infection by itself, however, does not affect the health status of an individual.
5. The induction of active TB is caused by an exaggerated inflammatory response against the bacilli. This has been explained as being induced by a high Th2 response and, more recently, by a strong neutrophilic reaction mediated by a Th17 response.
6. Therapeutic vaccines focused on bacilli-directed therapy are usually used as chemotherapy adjuvants and increase the Th1 response (Table 1).
7. Therapeutic vaccines focused on host-directed therapy can also be administered independently of chemotherapy and raise either a Th1 or a Treg response (Table 1).
8. All therapeutic vaccines can be given in different steps against Mtb infection, and have the advantage of not being conditioned by the drug sensitivity profile of Mtb.

**Table 1:** **Therapeutic vaccines against tuberculosis in clinical phase**.

**Vaccine**	**Mode of action**	**Mechanism of action**	**Antigens**	**References**
MIP	Bacilli-directed therapy	Th1 response	*M. avium* complex heat-killed whole bacilli	Das et al., 2015
RUTI		Th1 polyantigenic response	*M. tuberculosis* fragments	Nell et al., 2014
H56		Th1 response	ESAT-6; CFP-10; Rv2660c	Luabeya et al., 2015
*M. vaccae*/*obuense*	Host-directed therapy	Th1 response	Heat-killed whole bacilli	Lalvani et al., 2013
*M. manresensis*		Treg response		Montane et al., 2014

## Author contributions

The author confirms being the sole contributor of this work and approved it for publication.

### Conflict of interest statement

PC was one of the founders of Archivel Farma, the company that developed RUTI, and was its CSO until 2013. He was also the inventor of RUTI. PC is one of the founders and the CEO of Manremyc, the company that is developing the use of M. manresensis. He was also the inventor of this food supplement.
